# NOTCH1 signaling regulates the latent neurogenic program in adult reactive astrocytes after spinal cord injury

**DOI:** 10.7150/thno.71378

**Published:** 2022-05-27

**Authors:** Zijian Tan, Shangyao Qin, Yimin Yuan, Xin Hu, Xiao Huang, Hong Liu, Yingyan Pu, Cheng He, Zhida Su

**Affiliations:** Institute of Neuroscience, Key Laboratory of Molecular Neurobiology of Ministry of Education and the Collaborative Innovation Center for Brain Science, Naval Medical University, Shanghai 200433, China.

**Keywords:** NOTCH1, small molecule, astrocyte-to-neuron conversion, * in vivo* reprogramming, spinal cord injury

## Abstract

**Background:** Direct reprogramming of astrocytes into neurons opens up a new avenue for neuroregenerative medicine. However, the poor understanding of the molecular mechanisms underpinning the latent neurogenic program in astrocytes has largely restricted this strategy towards safe and effective clinical therapies.

**Methods:** Immunocytochemistry, immunohistochemistry, western blotting, qRT-PCR, gene knockdown and fate-mapping are performed to analyze the role of NOTCH1 signaling in regulation of the latent neurogenic program in reactive astrocytes after spinal cord injury.

**Results:** Western blotting analysis highlights that NOTCH1 is a key signaling mediating Ascl1- and Neurog2-driven astrocyte-to-neuron conversion. Inhibition of NOTCH1 signaling in cultured astrocytes by shRNA or DAPT (a NOTCH1 inhibitor) is sufficient to reprogram them into neurons by upregulating the expression of pro-neural transcription factors, including NeuroD1, NeuroD2, Pax6, Lmx1a and Lhx6. In the spinal cord of adult mouse, the expression of Notch1 is detected in resident astrocytes, which was significantly increased after spinal cord injury (SCI). Genetical knockdown of NOTCH1 signaling alone successfully triggers endogenous reactive astrocytes reprogramming into neurons in the injured adult spinal cord. Importantly, pharmacologically blocking NOTCH1 signaling with small molecule DAPT alone can also induce *in situ* astrocyte-to-neuron conversion after SCI.

**Conclusions:** We identify NOTCH1 as a key common signaling pathway in reactive astrocyte that provides a barrier for cell fate conversion. This proof-of-principle study will significantly expand our molecular understanding of astroglial-lineage reprogramming and overcoming the NOTCH1 gatekeeper with small molecules may provide a transgene-free approach for *in vivo* chemical neuronal reprogramming with potential clinical application in neuroregeneration.

## Introduction

In the adult mammalian central nervous system (CNS), most neurons fail to be replaced outside the neurogenic niches after injury or neurodegenerative disease. As a breakthrough in regenerative medicine, direct neuronal reprogramming holds great promise for CNS repair by providing patient-specific functional neurons. Specifically, *in vivo* neuronal reprogramming, which makes use of endogenous cells for generating functional new neurons, has been the focal point of ever-increasing interest and scrutiny. Massive strides have been made in this field in only a few short years 1-8; however, the poor understanding of the underlying molecular mechanism has greatly limited the development of neuronal reprogramming technology towards safe and effective clinical therapies.

Astrocytes are one of the major cell types that broadly distributed throughout the CNS, occupying 20-50% of brain volume in mammals. Reactive astrogliosis occurs as a prototypical response of CNS to injury or neurodegeneration 9-11. After CNS insults, resident astrocytes close to the lesion site become reactive and can proliferate to replenish themselves. Importantly, these activated astrocytes can even be dedifferentiated to acquire neural stem cell properties 12-14. Therefore, reactive astrocytes are regarded as an ideal cell source that is amenable to fate conversion 15. By forced expression of a single or a combination of pro-neural transcription factors, indeed, astrocytes have been successfully reprogrammed into different types of functional neurons *in vitro*, even *in vivo* 4-7, 16-21. However, little is known about the specific molecular mechanism underlying the direct lineage switching of astrocytes to induced neurons. During development, the generation of individual neuron types is in a hierarchical manner, in which a pan-neuronal identity is firstly induced before the neuron-type specific identity is finally committed by terminal selector transcription factors 22. In the process of astrocyte-to-neuron conversion mediated by different lineage-determining factors, whether there are common signaling mechanisms that instruct astrocytes to acquire a neuronal commitment remains an open question. Identifying these common signaling mechanisms may allow us to reprogram astrocytes into neurons by directly regulating the key signaling pathways with small molecules. Importantly, the small molecule-based chemical neuronal reprogramming does not require virus vector-mediated expression of exogenous transcription factors that may disrupt genomic integrity and raise safety concerns.

Ascl1 (achaete-scute homolog 1 or achaete-scute complex homolog 1) and Neurog2 (neurogenin-2) are expressed in stem/progenitor cells in the ventral and dorsal telencephalon and have been shown to regulate the specification of GABAergic and glutamatergic neurons during development, respectively 23-25. Recently, they have been widely used for direct neuronal reprogramming as key transcription factors. Forced expression of Ascl1 and Neurog2 in cultured astrocytes derived from cerebral cortex of postnatal mouse can accordingly instruct GAGAergic and glutamatergic neurons 17, 18. These two factors have also been shown to induce reactive astrocytes reprogramming into neurons in adult mouse brain 7, 19. In this study, using the cell model of Ascl1- and Neurog2-mediated direct astrocyte-to-neuron conversion, we identified NOTCH1 as a key common signaling pathway for astroglial-lineage reprogramming. *In vitro*, inhibition of NOTCH1 signaling by shRNA or DAPT resulted in upregulated expression of transcription factors, including NeuroD1, NeuroD2, Pax6, Lmx1a and Lhx6, in astrocytes and converted them into neurons. *In vivo*, the expression of NOTCH1 was detected in astrocytes, which was significantly increased after SCI. Importantly, genetically or pharmacologically blocking NOTCH1 signaling alone could successfully reprogram resident reactive astrocytes into neurons in the injured spinal cord of adult mouse.

In summary, we here show a NOTCH1-mediated cell fate safeguarding mechanism in astrocytes, which acts as an essential barrier for lineage conversion. Significantly, pharmacological regulation of NOTCH1 signaling may provide a transgene-free approach for *in vivo* neuronal reprogramming in a small molecule-based manner, holding the promise of future drug therapy for CNS repair.

## Materials and Methods

### Animals

Wild-type postnatal (P2-4) and adult (2-3 months old, female) C57/BL6J mice were purchased from Shanghai Ling Chang Biotech Co., Ltd. For genetic tracing of astrocytes, mGfap::Cre;Rosa::GFP transgenic mice obtained from Jackson Laboratory were used. Animals were housed in plastic cages on a normal 12 h light/dark cycle with food and water ad libitum. Animal procedures and protocols were approved by the Institutional Animal Care and Use Committee at Naval Medical University.

### Primary astrocyte culture

Highly enriched primary cortical postnatal and adult astrocytes were prepared as described previously 5, 26. Postnatal astrocytes were purified from P2-4 mouse brains and cultured in Dulbecco's Modified Eagle Medium (DMEM)/F12 (10% FBS, 1% penicillin/streptomycin), whereas adult astrocytes were obtained from 2-3 months old mouse brains and cultured in DMEM/F12 (20% FBS, 10 μM FSK, 10 ng/mL GDNF, 1% penicillin/streptomycin). After replaced with fresh culture medium on the next day, the medium was refreshed every 3 days. To obtain enriched astrocytes, loosely attached microglia and oligodendrocyte precursor cells were removed from the cell monolayer by shaking vigorously when cultured cells grew to confluence. All astrocytes subjected to *in vitro* reprogramming were passaged at least three times.

### Virus preparation and RNA Interference

Plasmid construction and lentivirus production were performed following previous protocols 5, 27. In brief, cDNAs encoding human Ascl1 or Neurog2 were subcloned into a third-generation lentiviral vector (pCSC-SP-IRES-GFP) to accordingly generate pCSC-SP-PW-Neurog2-IRES-GFP (abbreviated Neurog2) or pCSC-SP-PW-Ascl1-IRES-GFP (abbreviated Ascl1), in which the gene expression was under the control of a human GFAP promoter and the co-expressed GFP was used as a reporter. Replication-deficient lentivirus was generated in HEK293T cells by transient transfection with the lentiviral vector and packaging plasmids (pMDL, VSV-G, and pREV). For RNA interference, lentiviral vectors encoding Notch1 shRNA or a scramble shRNA were also prepared as above. The sequences for shRNA are listed below: scramble shRNA (5'-TTCTCCGAACGTGTCACGT-3' and 5'-ACGTGACACGTTCG GAGAA-3'), Notch1 shRNA (5'-CCGGTGGGCTATGAATTTCACCGTTTCAAGAGAACGGTGAAATTCATAGCCCTTTTTTG-3' and 5'-AATTCAAAAAAGGGCTATGAATTTCACCGTTCTCTTGAAACGGTGAAATTCATAGCCCA-3'). The scrambled shRNA was used as a control. The knockdown efficiency of Notch1 shRNA had been validated in our previous study 27.

### *In vitro* reprogramming

For *in vitro* astrocyte-to-neuron conversion, cultured astrocytes (4 x 10^4^/mL) were passaged and seeded on culture vessels or glass coverslips pre-coated with gelatin and matrigel. The next day, astrocytes were infected with Neurog2-, Ascl1- or Notch1 shRNA-expressing lentivirus or treated with NOTCH1 inhibitor DAPT. The following day, culture medium was switched to neuronal induction medium, DMEM:F12:neurobasal (2: 2: 1) containing 0.8% N-2 (Invitrogen) and 0.4% B-27 (Invitrogen). Then, the culture medium was half-changed every other day during the lineage reprogramming process. Immunostaining and morphological analysis were used to identify the astrocyte-converted neuronal cells.

### Western blotting and qRT-PCR

To detect the expression of signaling pathways, standard western blotting protocol was used. Cells were lysed in cell lysis buffer (50 mM Tris·HCl, pH 7.4, 150 mM NaCl, 1 mM EDTA and 1% Triton X-100) containing a mixture of protease (Roche) and phosphatase inhibitors (Sigma). Total proteins were collected, quantified and denatured by boiling in 2 × SDS loading buffer at 95 °C for 5min. Around 40 ug of protein was loaded per well and electrophoresed on 10% SDS-PAGE gel, and then transferred onto nitrocellulose membranes. Membranes were blocked with 5% nonfat milk and incubated with specific primary antibodies (Supplementary Table S1). After washing with buffer, membranes were incubated with horseradish peroxidase-conjugated secondary antibodies (Sigma; 1:10,000), and immunoreactive bands were visualized by chemiluminescence reagents (ECL, Amersham). Quantitative analysis of western blots was performed by densitometry and the values were pooled and expressed relative to GAPDH or β-ACTIN.

For gene expression analysis, qRT-PCR was performed. Total RNA was extracted from cultured cells with Trizol reagent (Invitrogen) and the contaminating DNA was removed with RNase-free DNase (Thermo Scientific Fermentas). RNA product was reverse transcribed into cDNA using RevertAid First Strand cDNA Synthesis Kit (Thermo Scientific Fermentas) according to the manufacture's instruction. PCR was performed using a MyiQ™ (Bio-Rad) with SYBR Green Realtime PCR Master Mix (TOYOBO Biotech). The gene expression was calculated and quantified by the 2^- ΔΔCt^ method and normalized to that of Gapdh. A detailed list of primers is available in Supplementary Table S2.

### Surgical procedures

To mimic clinical compression injury of spinal cord, a crush-injured SCI model was prepared with a pair of special forceps as previously reported protocol 28. Adult C57/BL6J mice (20-25 g) at 2-3 months of age were selected to ensure an identical physical size of spinal cords. To avoid the lesion variability that resulted from surgeons with different experience in surgical operations, all the crush SCI models were produced by the same surgeon. Briefly, animals were anesthetized with 2% pentobarbital (30 mg/kg) and subjected to a ~2 cm incision along the midline of the back. After laminectomy at T8, a pair of special forceps with a 0.4 mm spacer was used to laterally compress the spinal cord for 15 seconds to precisely produce a moderate crush wound. Then, animals were returned to their cages when they were fully awake, followed by manual bladder expression twice daily until recovery of reflexive bladder control. For postoperative pain control, animals were subcutaneously injected with buprenorphine (0.1 mg/kg). Spinal cords were collected at the indicated time and used for immunohistological analysis.

### Intraspinal viral injection

Using a Hamilton syringe and a 33 gauge, 45 degree-beveled needle (Hamilton, Reno, NV), 1.5 μL lentivirus (0.5-2×10^9^ cfu/mL) was manually injected into the spinal parenchyma at each of the two locations (1.5 mm proximal and distal to the lesion site) immediately after SCI. The virus was slowly injected within 1 min, and the needle was held at the injection site for 3 min and then slowly withdrawn within 1 min.

### DAPT and BrdU administration

DAPT (N-[N-(3,5-difluorophenacetyl)-l-alanyl]-S-phenylglycine t-butyl ester, Sigma) was dissolved in DMSO and diluted with PBS according to the manufacturer's instructions. For *in vitro* neuronal conversion, 10 μM DAPT was directly added into the cell cultures. For *in vivo* neuronal reprogramming, DAPT (10 mg kg^-1^ body weight) was administrated by intraperitoneal injection once a day.

BrdU (5-bromo-2-deoxyuridine, Sigma) was used to trace induced newborn neurons in the spinal cord. Animals were intraperitoneally injected with BrdU at a dose of 100 mg kg^-1^ body weight, twice daily for the indicated duration.

### Immunofluorescence

Immunocytochemistry was performed on adherent cells. After washed with PBS and fixed in 4% paraformaldehyde (PFA) for 20 min at room temperature, the cells were blocked and permeabilized with 3% BSA and 0.2% Triton X-100 in PBS for 1 hour, followed by staining with the indicated antibodies.

For immunohistochemistry, animals anesthetized and transcardiacally perfused with cold saline and 4% PFA. Spinal cords were dissected, post-fixed in 4% PFA overnight and cryoprotected by sinking in 30% sucrose at 4 °C for 48 h. The spinal sections were prepared by cutting on a cryostat (Leica) set at 14 μm thickness. After blocked and permeabilized with 3% BSA and 0.2% Triton X-100 in PBS, spinal sections were incubated in primary followed by secondary antibodies diluted in blocking solution.

For BrdU staining, spinal sections were incubated with 2 mol/L hydrochloric acid for 30 minutes at 37 °C. Then, the hydrochloric acid was washed off and the sections were incubated with 0.1 mol/L sodium tetraborate for 5 minutes twice. Finally, BrdU incorporation was detected by fluorescent staining using an anti-BrdU antibody.

During the immunostaining analysis, cell nuclei were counterstained with Hoechst 33342 (Hst). Images were captured with a fluorescence microscope (Nikon E660FN) or a confocal laserscanning microscope (Leica SP5). The subsequent processing of images was performed by Image-Pro Plus and Adobe Photoshop CS5. A detailed list of antibodies is available in Supplementary Table S3.

### Data and statistical analysis

All results were validated by at least three mice or independent experiments. Data collection and analyses were performed by experiments blind to the treatment conditions. The quantitative data were plotted as mean ± s.d and statistical analysis was performed using Student's *t*-test or one-way ANOVA with Tukey's *post hoc* test. Differences were considered statistically significant at P < 0.05.

## Results

### Identification of NOTCH1 as an essential signaling in astrocytes for neuronal reprogramming

**Figure 1:**
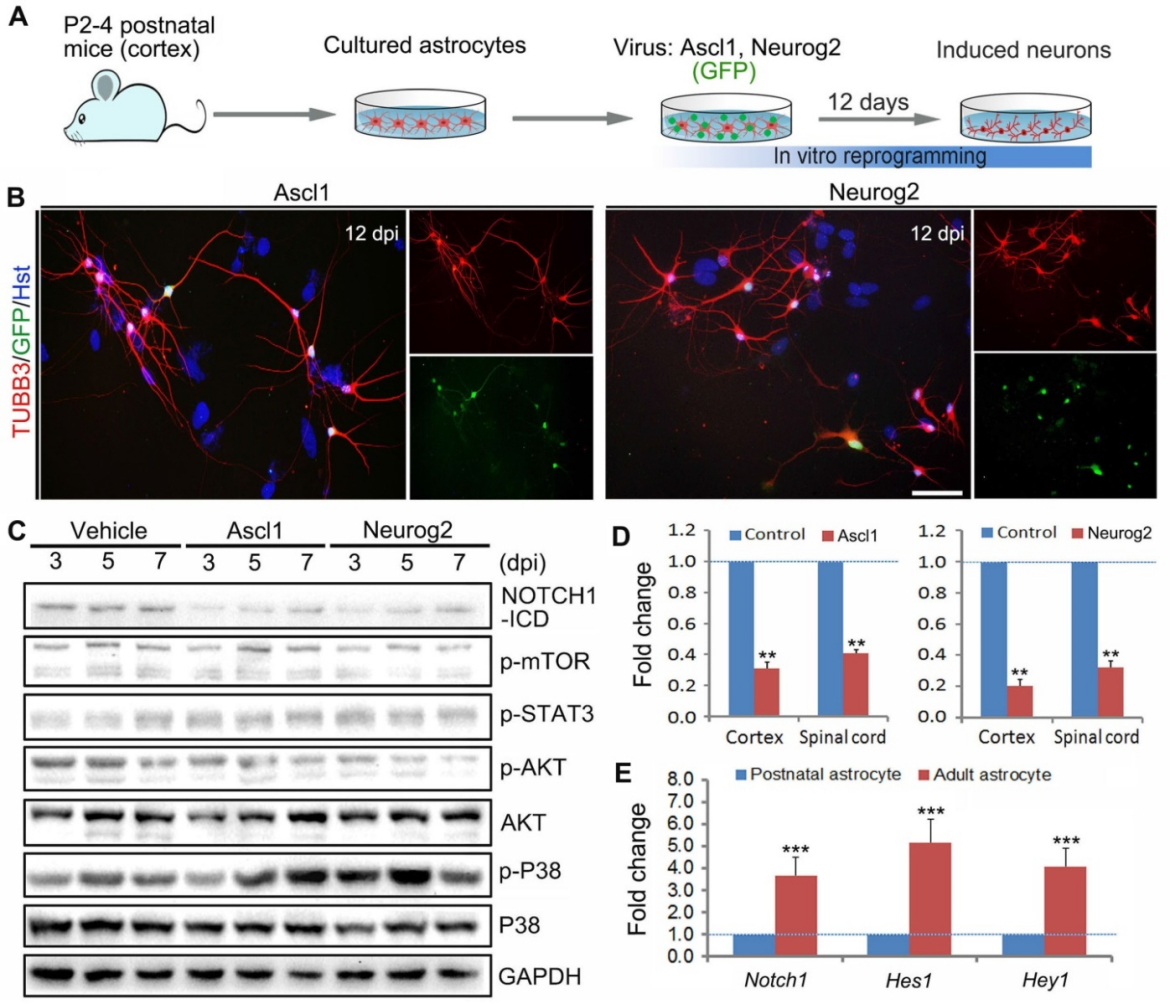
** Identification of essential signaling pathways in astrocyte reprogramming. (A)** Experimental procedures. **(B)** Representative images of Ascl1 or Neurog2-induced neurons from postnatal cortical astrocytes by staining with Tuj1 at 12 dpi. Nuclei were counterstained with Hoechst 33342 (Hst).** (C)** Western blotting analysis of signaling pathways, including NOTCH1, mTOR, STAT3, AKT and P38, in Ascl1 or Neurog2-mediated astrocyte-to-neuron conversion. **(D)** qRT-PCR analysis of Notch1 expression in postnatal cortical and spinal astrocytes infected with Ascl1- or Neurog2-expressing virus at 3 dpi. **(E)** The expression of Notch1 signaling molecules, Notch1, Hes1 and Hey 1, in postnatal and adult cortical astrocytes was determined by qRT-PCR. **p < 0.01, ***p < 0.001 by Student's *t*-test (n = 3 for each group). The scale bar represents 50 µm.

In this study, we aimed at investigating whether there are common signaling mechanisms underlying different transcription factors-mediated direct astrocyte-to-neuron conversion. Highly enriched primary astrocytes were isolated from cerebral cortex of newborn mouse (Figure 1A). The purity of cultured astrocytes was determined by immunocytochemical analysis with various astrocytic markers in our previous study 27. As shown in Supplementary Figure S1, neither neurons (immunoreactive for DCX, TUBB3 or MAP2) nor neural stem cells (NESTIN^+^) were detected in these cultures. The cDNA of human Ascl1 and Neurog2 was sub-cloned into a lentiviral construct, respectively, in which the gene expression is driven by a GFAP promoter and the co-expressed GFP is used to visualize virus-infected cells. Consistent with previous studies 17, 18, 27, when transduced with lentivirus expressing Ascl1 or Neurog2, the cultured astrocytes were successfully reprogrammed into neurons, as assessed by morphology and TUBB3 immunostaining (Figure 1 A and B). At 12 days post infection (dpi), Ascl1 and Neurog2 shared a similar neuronal induction efficiency, about 50% of GFP^+^ cells (data not shown).

Using the cell model of Ascl1- and Neurog2-mediated direct neuronal reprogramming, we performed western blotting to identify the common signaling mechanisms underlying astrocyte-to-neuron conversion. After cultured astrocytes were infected with Ascl1- and Neurog2-expressing virus or control virus (Vehicle), the proteins were collected at 3, 5 and 7 dpi for western blotting analysis of signaling pathways, including NOTCH1, mTOR, STAT3, AKT and P38. Figure 1C showed that forced expression of Ascl1 or Neurog2 in astrocytes both resulted in a dramatic decrease of NOTCH1-ICD (NOTCH1 intracellular domain), whereas there was no significant effect on mTOR, STAT3, and P38. Of note, although ectopic expression of Neurog2 significantly reduced the expression level of p-AKT in astrocytes, this phenomenon was not observed in the Ascl1-treated astrocytes (Figure 1C). The decreased expression of NOTCH1 in Ascl1- or Neurog2-infected astrocytes was further confirmed by qRT-PCR (Figure 1D). Together, these findings suggest that NOTCH1 may be an essential signaling pathway that provides a barrier for astrocyte-to-neuron conversion. Our previous study indicated that neuronal conversion is relatively inefficient in adult astrocytes compared to that in postnatal astrocytes 27. Interestingly, we found that the expression of Notch1 and its target genes (Hes1 and Hey1) was significantly higher in adult astrocytes than that in postnatal astrocytes (Figure 1E), further highlighting that NOTCH1 is a common signaling mediating Ascl1- and Neurog2-driven astrocyte-to-neuron conversion, in which it may function as a repressor of lineage reprogramming.

### NOTCH1 inhibition successfully reprograms astrocytes into neurons *in vitro*

**Figure 2:**
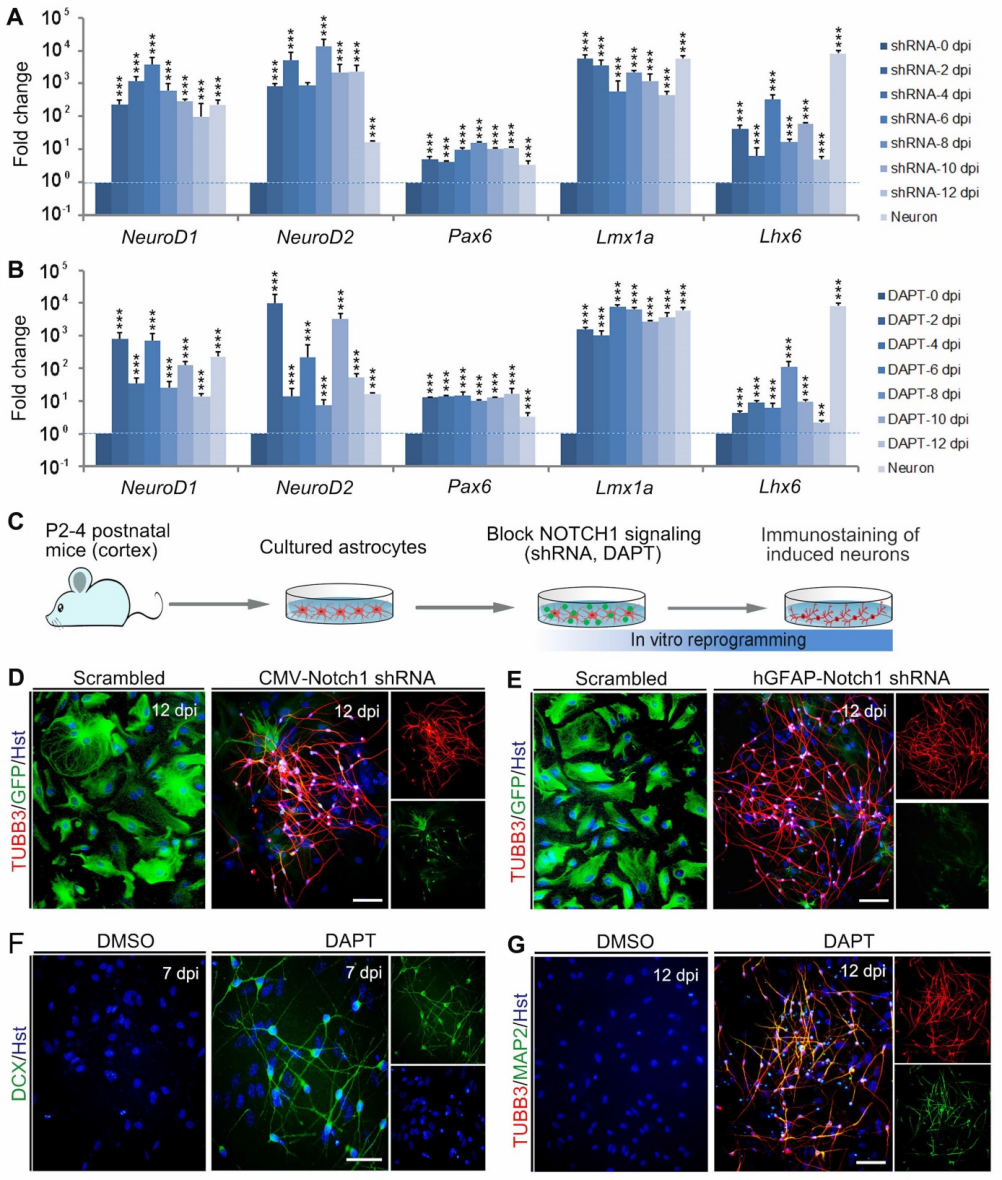
** Reprogramming astrocytes into neurons in response to Notch1 inhibition in vitro. (A and B)** After Notch1 was inhibited by shRNA (A) or DAPT (B), the expression of representative genes for neurogenic factors in astrocytes was determined by qRT-PCR analysis at the indicated time. The expression of these neurogenic factors in neurons was served as a positive control. **(C)** Experimental procedures. **(D and E)** Notch1 knockdown by shRNA induced astroyctes reprogramming into neurons. **(F and G)** Pharmacological inhibition of Notch1 by DAPT induced astroyctes reprogramming into neurons. **p < 0.01, ***p < 0.001 by one-way ANOVA with Tukey's *post hoc* test (n = 3 for each group). The scale bars represent 50 µm.

Based on its critical role in neuronal reprogramming, we next investigated whether de-repression of NOTCH1 could directly convert astrocytes into neurons. Initially, a short hairpin RNA (shRNA) constructed in lentiviral vector (CMV-Notch1 shRNA) was used to knock down Notch1. The activity of this shRNA had been validated in our previous study 27. After treatment of astrocytes with the Notch1 shRNA, qRT-PCR was performed to analyze the expression of pro-neural transcription factors in astrocytes at 0, 2, 4, 6, 8, 10 and 12 dpi. As shown in Figure 2A, knockdown of Notch1 by shRNA resulted in a significant increase in the expression of neurogenetic transcription factors, including NeuroD1, NeuroD2, Pax6, Lmx1a and Lhx6. For canonical NOTCH1 signaling, the NOTCH1 transmembrane domain is cleaved by γ-secretase to release the NOTCH1-ICD, which can translocate to the nucleus and modulate transcription 29. DAPT (N-[N-(3,5-difluorophenacetyl)-l-alanyl]-S-phenylglycine t-butyl ester) is a γ-secretase inhibitor that can inhibit the generation of NOTCH1-ICD to block the NOTCH1 signaling pathway 30, 31. Therefore, we then treated the cultured astrocytes with DAPT to inhibit NOTCH1 signaling. Similarly, qRT-PCR analysis revealed that the pro-neural transcription factors, NeuroD1, NeuroD2, Pax6, Lmx1a and Lhx6, were also markedly upregulated by DAPT treatment at the indicated time points (Figure 2B).

To determine whether Notch1 knockdown alone could elicit neuronal reprogramming, cultured astrocytes were infected with lentivirus expressing Notch1 shRNA (CMV-Notch1 shRNA, Figure 2C). Interestingly, we found that Notch1 knockdown caused obvious changes in cell morphology of astrocytes, rapidly losing their flat morphology and adopting a typical neuronal morphology with long neurites (Figure 2D). In sharp contrast, no significant change of cell morphology was observed in the control virus-infected astrocytes (Figure 2D). Consistent with the morphological changes, immunocytochemical analysis showed that these cells became immunoreactive for the pan-neuronal marker TUBB3 at 12 dpi (Figure 2D). To specifically target astrocytes, the Notch1 shRNA was sub-cloned into a lentiviral vector where gene expression was driven by a human GFAP promoter (hGFAP-Notch1 shRNA). Similarly, astrocytes infected with hGFAP-Notch1 shRNA could also be successfully converted into TUBB3-positive neurons (Figure 2 C and E).

Although the transcription factor-mediated neuronal reprogramming technology has grown exponentially in the past few years, it raises the safety concerns for future clinical applications, such as the risks of integrating exogenous DNA and reactivation of exogenous genetic factors 32, 33. To circumvent the concerns, small molecule-based cellular fate reprogramming was introduced as an alternative transgene-free approach. Therefore, we further tested whether chemically regulating NOTCH1 signaling could directly induce astrocytes-to-neuron conversion (Figure 2C). After treated with the chemical compound DAPT, immunostaining revealed that cultured astrocytes were also successfully reprogrammed into DCX-positive neuroblasts at 7 dpi (Figure 2F). These DAPT-induced cells were shown to express pan-neuronal marker TUBB3 and mature neuronal marker MAP2 at 12 dpi (Figure 2G). Of note, treatment of astrocytes with AKT inhibitor IV (10 µM, Merck Calbiochem) failed to reprogram them into neurons (Supplementary Figure S2). Taken together, these results indicate that blocking NOTCH1 alone can elicit endogenous neurogenic program in astrocytes which is sufficient to efficiently reprogram them into neuronal cells.

### Genetical knockdown of NOTCH1 signaling successfully reprograms resident astrocytes into neurons in injured adult spinal cord

**Figure 3:**
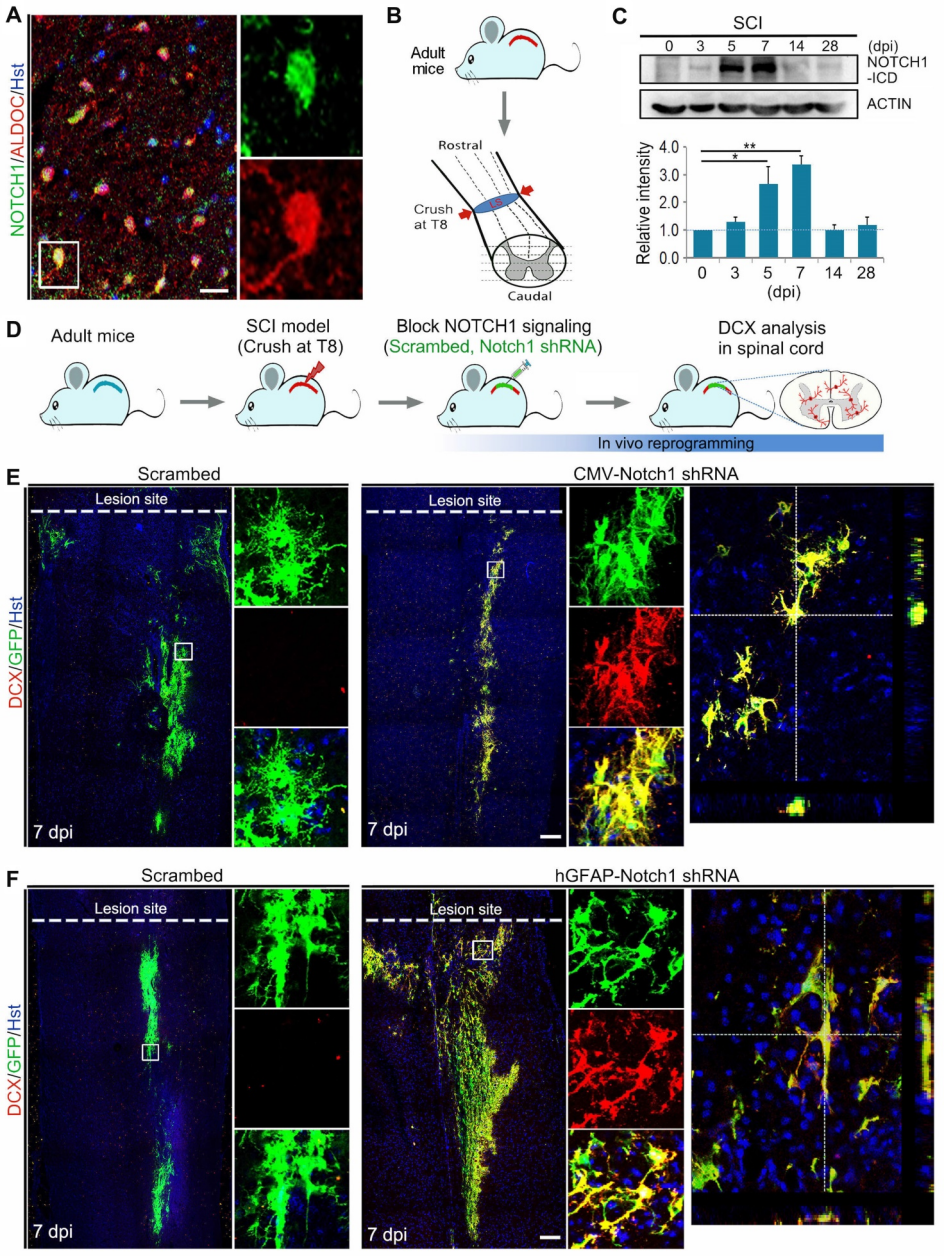
** Notch1 knockdown in spinal astrocytes gives rise to neurogenesis in the injured adult spinal cord. (A)** Immunohistochemical analysis of Notch1 expression in astrocytes in intact spinal cord. **(B)** Schematic diagrams of SCI model. Spinal cord was crushed at T8 level. LS, lesion site. **(C)** Western blotting analysis of NOTCH1 expression in spinal cord at 0, 3, 5, 7, 14 and 28 days post injury (dpi). **(D)** Experimental procedures. **(E and F)** shRNA-mediated Notch1 knockdown in astroyctes triggered neuroblast production in crush-injured spinal cord. Orthogonal views of cells with expression of the DCX and GFP are also shown in right panel. *p < 0.05, **p < 0.01 by one-way ANOVA with Tukey's *post hoc* test (n = 3 for each group). The scale bars represent 50 µm (A) and 200 µm (E and F).

In order to investigate whether the inhibition of NOTCH1 signaling alone could induce neuronal reprogramming *in vivo*, we first performed immunohistochemical analysis on the expression of NOTCH1 in spinal astrocytes of adult mouse. As shown in Figure 3A, NOTCH1 was detectable in ALDOC^+^ astrocytes in the intact spinal cord. We next set up a clinically relevant SCI model in which traumatic injury was induced by a crush wound at T8 level of spinal cord (Figure 3B). Interestingly, NOTCH1 expression was also detected in reactive astrocytes in the injured spinal cord (Supplementary Figure S3). Importantly, western blotting showed that the expression level of NOTCH1 in spinal cord was dramatically upregulated after SCI, peaking at 7 dpi (Figure 3C). In the injured spinal cord, it is of note that strong staining signals were detected in cell nuclei, suggestive of activation of NOTCH1 signaling (Supplementary Figure S3).

Using the SCI model, we injected lentivirus expressing Notch1 shRNA into the parenchyma of injured adult spinal cord immediately after SCI and neurogenesis was analyzed by DCX immunostaining at 7 dpi (Figure 3D, Supplementary Figure S4A). Histological analysis of longitudinal sections spanning the lesion site showed that virus-transduced cells (indicated by GFP^+^) were detectable in a broad area around the injection site both rostrally and caudally (Supplementary Figure S4B). Interestingly, we observed that DCX-positive neuroblasts were induced in the crush-injured spinal cord injected with lentivirus expressing either CMV-Notch1 shRNA or hGFAP-Notch1 shRNA at 7 dpi, while no DCX signal was detectable in that injected with control virus (Figure 3 E and F). All the induced DCX-positive cells were mainly distributed around the injection site and were co-labelled by GFP, indicative of an origin from virus-transduced cells (Figure 3 E and F). As shown in Supplementary Figure S5, the DCX^+^ cells could be detected in the lesioned spinal cord injected with hGFAP-Notch1 shRNA virus even at 2 and 4 weeks post injury (wpi). Of note, these DCX-positive cells were shown to incorporate BrdU, suggesting that they might be newly generated neurons from proliferative astrocytes (Supplementary Figure S6). Together, these data provide evidence that Notch1 knockdown alone can successfully give rise to neurogenesis in an injured environment of the adult spinal cord.

### Pharmacological inhibition of NOTCH1 signaling successfully elicits neurogenesis in injured adult spinal cord

**Figure 4:**
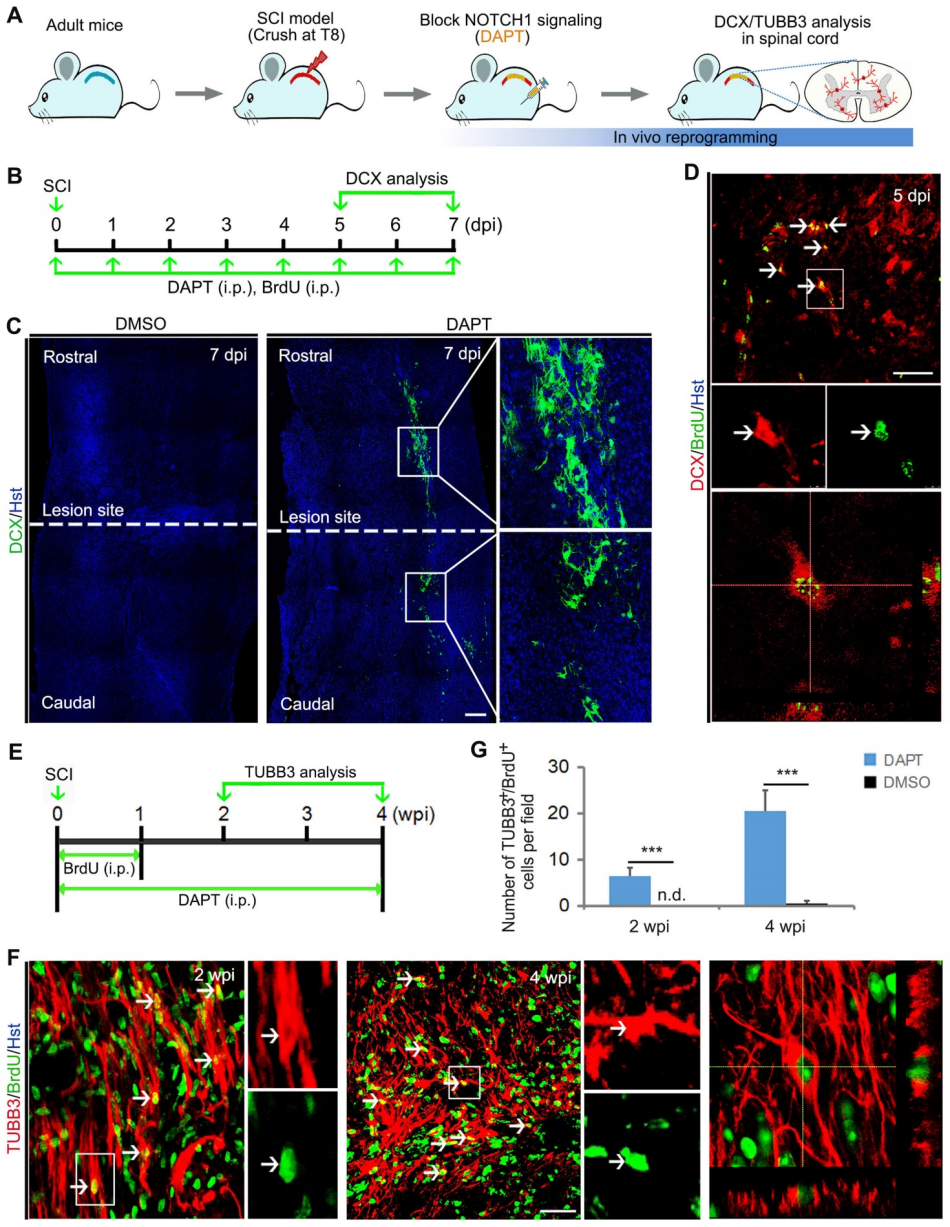
** Pharmacological inhibition of Notch1 signaling gives rise to neurogenesis in the injured adult spinal cord. (A)** Experimental procedures. **(B)** Schematic diagrams of study design for (C) and (D). LS, lesion site. i.p., intraperitoneal injection. **(C)** DAPT-mediated pharmacological inhibition of Notch1 signaling triggered neuroblast production in crush-injured spinal cord. **(D)** Incorporation of BrdU in induced DCX-positive cells. Arrows indicate the BrdU^+^/DCX^+^ cells. An orthogonal view of BrdU^+^/DCX^+^ cells is shown in the bottom panel. **(E)** Schematic diagrams of study design for (F). i.p., intraperitoneal injection. **(F)** Representative images of newly generated neurons indicated by BrdU-traced TUBB3 positive cells in the injured adult spinal cord of animal injected with DAPT at 2 and 4 wpi. Arrows indicate the BrdU^+^/TUBB3^+^ cells. Orthogonal view of cells with expression of the TUBB3 and BrdU is also shown in the right panel. **(G)** Quantification of DAPT-induced BrdU^+^/TUBB3^+^ cells in the injured adult spinal cord at 2 and 4 wpi. ***P < 0.001 by Student's *t*-test (n = 5 mice per group; n.d., no detected). The scale bars represent 200 µm (C) and 50 µm (D and F).

To examine whether chemically blocking NOTCH1 signaling could elicit neurogenesis in the injured adult spinal cord, animals with SCI were administrated with the NOTCH1 signaling inhibitor DAPT by intraperitoneal injection (Figure 4 A and B).

In the meantime, these animals were also intraperitoneally injected with DNA base analog BrdU to trace the induced newborn neurons in the injured spinal cord (Figure 4B). Similar to Notch1 shRNA, we found that chemical compound DAPT-mediated blockade of NOTCH1 signaling also triggered DCX-positive neuroblast production around the lesion site in the injured spinal cord (Figure 4C). In sharp contrast, DCX signal was not detected in DMSO-injected spinal cord (Figure 4C). Importantly, these DCX^+^ cells were shown to incorporate BrdU, suggesting that they were newly generated neurons (Figure 4D). In addition, the DAPT-induced neurogenesis was further identified by TUBB3 staining (Figure 4 A and E). At 2 and 4 weeks post injury (wpi), immunochemical analysis revealed that BrdU-traced TUBB3 positive cells were detected in the crush-injured spinal cord of animals injected with DAPT (Figure 4 F and G).

**Figure 5:**
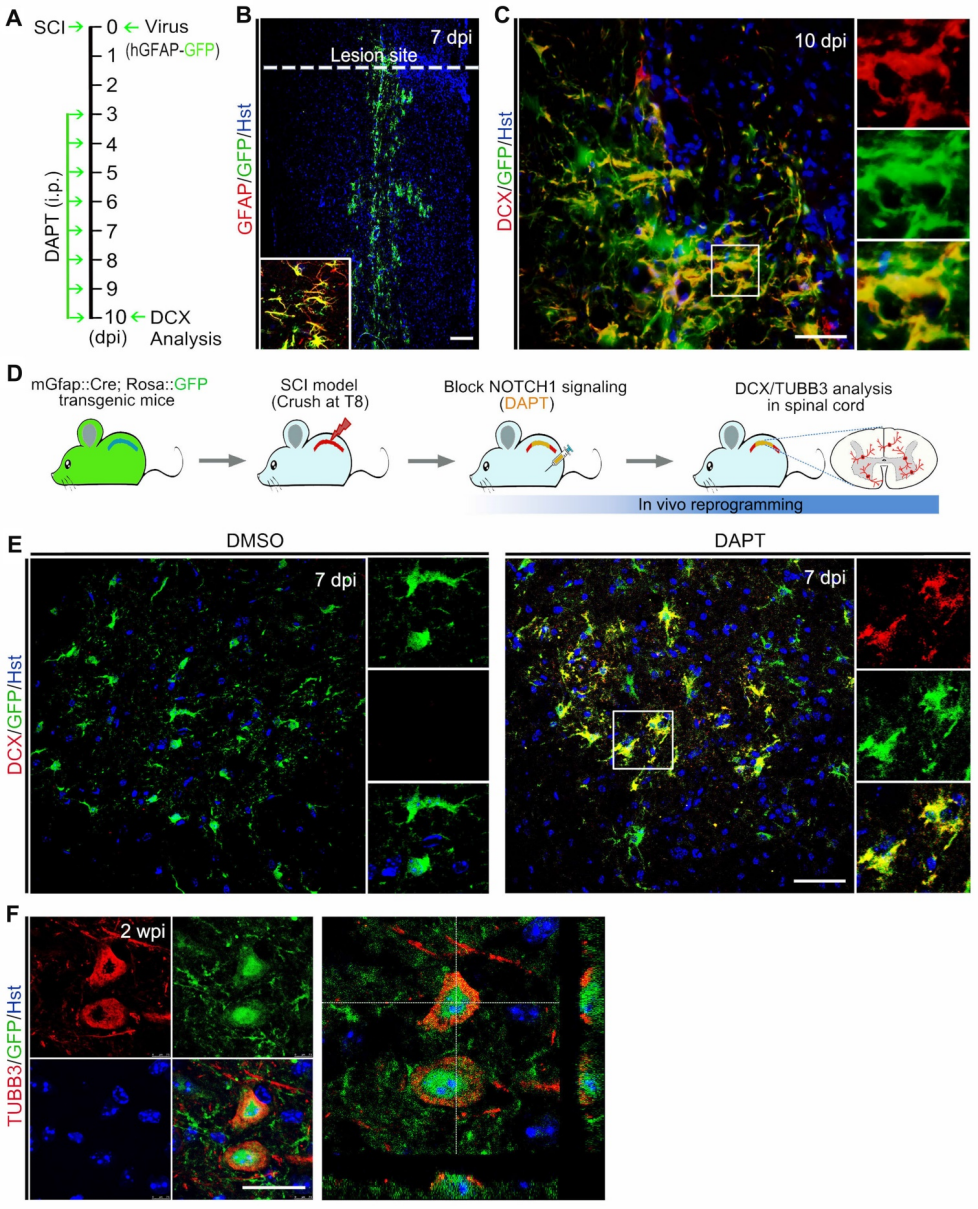
** Identifying the cellular origin of DAPT-induced new neurons. (A)** Schematic diagrams of study design for (B) and (C). **(B and C)** Tracing DAPT-induced new neurons by injection of spinal cord with hGFAP-GFP lentivirus. (B) A lower magnification view of a longitudinal section from the injured adult spinal cord injected with hGFAP-GFP lentivirus at 7 dpi. Confocal analysis showed that hGFAP-GFP lentivirus specifically targeted GFAP^+^ astrocytes. (C) Representative images showing that DAPT-induced DCX^+^ cells originated from virus-infected astrocytes (indicated by GFP^+^). **(D)** Experimental procedures for (E) and (F). **(E and F)** Fate-mapping analysis of DAPT-induced new neurons with mGfap::Cre; Rosa::GFP transgenic mice. Immunohistochemical analysis showed that DAPT-induced DCX^+^ and TUBB3^+^ cells were derived from GFP-traced spinal astrocytes. The scale bars represent 100 µm (B), 50 µm (C and E), 25 µm (F).

Because DAPT does not specifically target spinal astrocytes, we performed fate-mapping analysis to investigate whether the DAPT-induced newborn neurons came from resident spinal astrocytes. Initially, the astrocytic origin of newly generated neurons was confirmed by viral tracing. Lentivirus expressing hGFAP-GFP was injected into the spinal cord immediately after crush wounding, followed by DAPT administration for 8 consecutive days from 3 to 10 dpi (Figure 5A). As shown in Figure 5B, GFAP^+^ resident astrocytes were specifically traced by this virus. At 10 dpi, immunostaining showed that DCX-positive cells were induced in the injured spinal cord with a majority of them co-labelled by GFP, suggesting that they originated from spinal astrocytes (Figure 5C). Additionally, the cellular source for DAPT-induced newborn neurons was also determined by genetic lineage tracing. Supplementary Figure S7A revealed that Cre was exclusively expressed in GFAP-positive astrocytes in the spinal cord of mGfap::Cre transgenic mice. Then, we crossed this line to Rosa::GFP reporter to generate mGfap::Cre;Rosa::GFP transgenic mice in which astrocytes are specifically labelled by GFP (Supplementary Figure S7B). Adult mGfap::Cre;Rosa::GFP transgenic mice were subjected to spinal crush wound and received DAPT treatment (Figure 5D). Immunohistochemical analysis showed that a fraction of GFP-traced cells were co-labelled by the neuronal marker DCX in the spinal cord of DAPT-treated mice at 7 dpi, whereas no DCX signal was detected in the spinal cord of DMSO-treated mice (Figure 5E). At 2 wpi, DAPT treatment also induced TUBB3^+^/GFP^+^ cells in the spinal cord (Figure 5F). Collectively, these findings provide evidence that DAPT-induced neurogenesis originates from spinal astrocytes.

### Maturation of chemical-induced new neurons in the injured adult spinal cord

**Figure 6:**
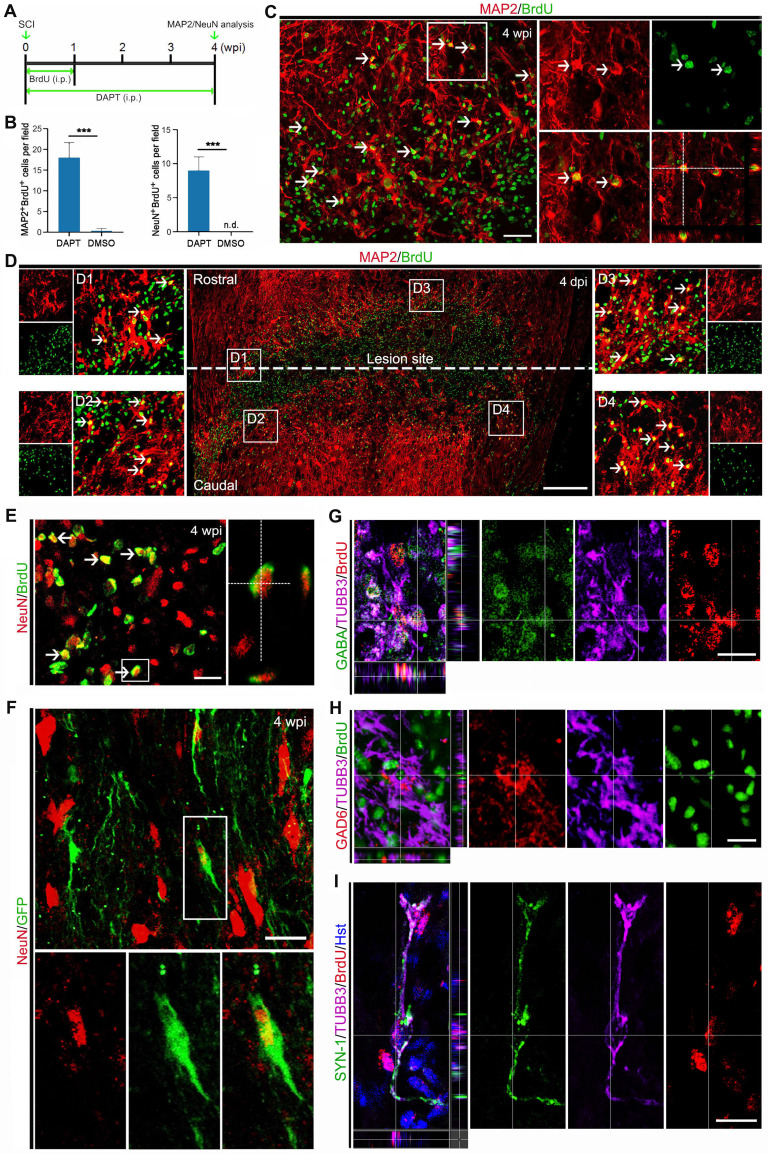
** Maturation of DAPT-induced new neurons in the injured adult spinal cord. (A)** Schematic diagram of study design for (B-E). i.p., intraperitoneal injection. **(B-E)** Expression of the mature neuronal markers MAP2 (C and D) or NeuN (E) in BrdU-traced cells in the injured adult spinal cord of animals injected with DAPT at 4 wpi. MAP2^+^/BrdU^+^ and NeuN^+^/BrdU^+^ cells are indicated by arrowheads. Note that MAP2^+^/BrdU^+^ cells are located around the lesion site (D). Orthogonal views of cells with expression of the MAP2/BrdU and NeuN/BrdU are also shown in (C) and (E), respectively. ***P < 0.001 by Student's *t*-test (n = 5 mice per group). **(F)** Representative images of NeuN^+^/GFP^+^ cells in the injured adult spinal cord of mGfap::Cre; Rosa::GFP transgenic mice injected with DAPT at 4 wpi. **(G-I)** Confocal images show that DAPT-induced newborn neurons (indicated by BrdU^+^/TUBB3^+^) express GABA, GAD6 and SYN-1 at 4 wpi. The scale bars represent 50 µm (C), 200 µm (D) and 25 µm (E-I).

To determine whether the DAPT-induced new neurons in the injured spinal cord can develop into mature neurons, their cellular fate was then evaluated by BrdU incorporation and immunostaining with the mature neuron-specific markers, MAP2 and NeuN (Figure 6A). When examined at 4 wpi, 17.80 ± 4.94 MAP2-positive cells per field were co-labelled by BrdU (Figure 6 B and C). It is of note that these DAPT-induced MAP2^+^/BrdU^+^ cells were mainly distributed around the lesion site (Figure 6D). Correspondingly, an estimated 9.08 ± 2.67 NeuN-positive cells per field were also shown to incorporate BrdU (Figure 6 B and E). These BrdU-incorprated MAP2- or NeuN-positive cells provided evidence that they were mature neurons reprogrammed through cell division. In stark contrast, few MAP2 and NeuN were detected in BrdU-traced cells in the spinal cord treated with DMSO (Figure 6B). In addition, the mGfap::Cre;Rosa::GFP transgenic mice were also used to genetically trace DAPT-induced mature neurons. Immunohistochemical analysis revealed that a fraction of GFP-positive cells was immunoreactive for NeuN at 4 wpi, suggestive of astrocyte-converted mature neurons (Figure 6F). Together, these data indicate that DAPT-induced neurons can become mature in the injured adult spinal cord.

The cellular identity of DAPT-induced new neurons was further analyzed by immunohistochemistry. After DAPT injection, the induced newborn neurons were indicated by BrdU^+^/TUBB3^+^ cells in the injured spinal cord (Figure 6 G-I). We did not find any DAPT-induced newborn neurons expressing choline acetyltransferase (ChAT, a marker for cholinergic motor neurons) or vesicular glutamate transporter 1 (VGLUT-1, a marker for excitatory neurons) (data not shown). In contrast, these newly generated neurons were observed to express GABA (Gamma aminobutyric acid) and GAD6 (Glutamic acid decarboxylase 65/67), markers for inhibitory neurons (Figure 6 G and H). Importantly, some DAPT-induced newborn neurons were co-labelled by synapsin-1 (SYN1), a marker for presynaptic terminals (Figure 6I). Together, these results suggest that DAPT-mediated inhibition of Notch1 signaling can convert local reactive astrocytes into synapse-forming GABAergic interneurons in the injured adult spinal cord.

## Discussion

In response to diverse types of CNS injury, resident astrocytes become reactive and proliferative, and subpopulations of them are shown to acquire neural stem cell properties, which may provide an important new resource for endogenous repair 12-14. Of note, albeit the reactive astrocytes isolated from injured CNS reveal their multipotency and capacity for self-renewal *in vitro*, they remain within their lineage and fail to produce neurons *in vivo* 12, 34. Therefore, identifying the signals that retain reactive astrocytes in their lineage will significantly contribute to relieving them from this restriction. Here, our proof-of-principle study demonstrated that NOTCH1 signaling pathway in reactive astrocyte represented a key barrier in neuronal reprogramming. Importantly, chemical compound-based inhibition of NOTCH1 signaling successfully reprogrammed reactive astrocytes into neurons in the crush-injured adult spinal cord, shedding light on the potential clinical application in CNS repair.

Because astrocytes are widely distributed in the CNS and retain some of the original patterning information from their radial glial ancestors 35, they are regarded as ideal candidates for neuronal reprogramming. A number of transcription factors have been shown to successfully reprogram astrocytes into neurons *in vitro* or *in vivo* 4-7, 16-21, while the underlying mechanisms remain poorly understood. Transcriptional analysis at early stages of Ascl1- and Neurog2-mediated* in vitro* direct reprogramming of murine postnatal astrocytes into neurons showed that Ascl1 and Neurog2 rapidly elicited distinct neurogenic programs with only a small subset of shared target genes 36. By western blotting and qRT-PCR analyses, we here found that NOTCH1 was an essential signaling pathway commonly regulated by Ascl1 and Neurog2 during reprogramming cultured astrocytes into neurons. NOTCH1 was markedly down-regulated upon forced expression of Ascl1 or Neurog2 in astrocytes. In our previous study, we showed that blocking of NOTCH1 signaling can significantly increase the efficiency of Ascl1- or Neurog2-mediated neuronal reprogramming from cultured astrocytes 27. In the present study, strikingly, we observed that RNA interference or DAPT-mediated NOTCH1 inhibition alone was sufficient to elicit neuronal reprogramming from astrocytes *in vitro*. All these data suggest that NOTCH1 signaling in astrocytes functions as a key barrier in direct neuronal reprogramming.

After SCI, irreversible neuronal loss often leads to disastrous functional impairments and induction of endogenous neuroregeneration provides a promising strategy for spinal cord repair. Although reactive astrocytes in the injured spinal cord take on an NSC character, they fail to produce neurons* in vivo*. The *in vitro* findings prompted us to investigate whether NOTCH1 signaling also represented a repressor that restricted reactive astrocytes in their lineage. By immunohistochemistry and western blotting analysis, we showed that NOTCH1 expression was detected in resident astrocytes in intact spinal cord of adult mouse and enhanced in response to SCI, consistent with previous results in adult rat spinal cord 37. Importantly, strong staining signals were observed in cell nuclei, providing evidence that the NOTCH1 signaling was activated in reactive astrocytes. When NOTCH1 in reactive astrocytes was knocked down by shRNA or inhibited by small molecule DAPT, DCX-positive neuroblasts were successfully induced in the damaged adult spinal cord. Fate-mapping analysis confirmed that these newborn neuroblasts were derived from resident spinal astrocytes. Furthermore, immunohistochemical analysis revealed that they could develop into mature neurons in the local environment of SCI. These findings indicate that NOTCH1 is also a key repressor of* in vivo* lineage conversion and high level of NOTCH1 signaling in reactive astrocytes make them refractory to neuronal reprogramming. Of note, although reactive astrocytes can be converted into neurons by NOTCH1 signaling blocking alone, the reprogramming efficiency is low in our current experimental conditions. Further optimization of the reprogramming strategy is necessary to generate more neurons that are required for SCI repair. In addition, future work is also required to examine the subtype of induced neurons and to confirm whether they are functional for repairing the damaged spinal cord.

NOTCH pathway is a highly conserved signaling system that plays essential roles in cell-fate specification, differentiation, and developmental patterning 38. For example, NOTCH signaling inhibits an early differentiation step of progenitors by suppression of neurogenic basic helix-loop-helix (bHLH) factors, such as Ascl1 and Neurog2 37, 39. Attenuation of NOTCH signaling is shown to enhance neurogenesis 37. In our study, although the specific molecular mechanisms of the NOTCH1 blocking-driven astrocyte-to-neuron conversion are unclear, we found that endogenous neural transcription factors, including NeuroD1/2, Pax6, Lmx1a and Lhx6, were activated during the lineage conversion. As a member of the bHLH transcription factor family, NeuroD1 is a key regulator of the fate of specific neuronal cells and plays an essential role in adult neurogenesis 40-42. Ectopic expression of NeuroD1 in astrocytes has been shown to successfully induce neuronal cell fate *in vitro* and *in vivo* 6, 20. NeuroD2, another bHLH transcription factor, is an early-onset neuronal transcript required for the development and survival of CNS neurons 43. As a neurogenesis-controlling factor, Pax6 is shown to function as intrinsic fate determinant of the neurogenic potential of glial cells 44. In addition, both Lmx1a and Lhx6 are also neuron-fate-determining proneural genes that have been used to induce neuronal reprogramming 16, 45. Together with these previous studies, our data highlight that these activated neural transcription factors may play critical roles in the astrocyte-to-neuron conversion induced by NOTCH1 inhibition. NOTCH is a single-pass transmembrane receptor. In the canonical pathway, NOTCH is activated by ligands on adjacent cells and cleaved by a γ-secretase enzyme complex, releasing a NOTCH1-ICD that is translocated into the nucleus, where it leads to transcriptional activation of target genes 46. The functions of NOTCH are mainly mediated through the canonical pathway, while a non-canonical NOTCH pathway (ligand- or transcription-independent) has also been reported 46. For instance, NOTCH inhibition is shown to enhance cardiac reprogramming by increasing the binding of transcription factor MEF2C to the promoter regions of cardiac structural genes, whereas none of the most common downstream target genes of the canonical NOTCH pathway, including the *Hes*/*Hey* gene family, are significantly down-regulated, suggestive of a mechanism mediated by non-canonical NOTCH pathway 47. In our study, we found that *Hes1* and *Hey1* genes were highly expressed in adult astrocytes. Inhibition of NOTCH1 signaling by the γ-secretase inhibitor DAPT resulted in a significant decrease of the expression of Hes1 and Hey1 during the direct astrocyte-to-neuron conversion (data not shown). Together, these findings suggest that NOTCH1 inhibition reprograms astrocytes into neurons via the canonical pathway, although other mechanisms may also contribute to this process.

In recent years, small molecules have emerged as promising strategies for cell fate conversion 32. Compared with transgenic approaches, small molecules have shown their power and advantages in reprogramming, which can circumvent the safe concerns of conventional transgenic-based lineage conversion, including the risk of introduction of foreign genetic material and reactivation of exogenous genetic factors 48-51. Therefore, chemical compound-driven direct cell fate reprogramming, especially* in vivo*, has represented a potential new strategy for regenerative therapy. In a recent study, interestingly, a chemical cocktail with five small molecules was shown to reprogram endogenous astrocytes into neurons in the intact adult brain, including striatum and cortex 52. However, whether small molecules can induce neuronal reprogramming in the adult spinal cord remains unknown, especially in the case of SCI. Here, our study provides an important clue that chemically targeting the NOTCH1 signaling pathway in reactive astrocytes may be an especially suitable approach for *in situ* neuronal reprogramming in the damaged spinal cord. In fact, inhibition of NOTCH1 signaling by the small molecule DAPT successfully reprogram resident spinal reactive astrocytes into neurons after SCI. Of note, NOTCH inhibitors are currently in several preclinical and clinical trials for cancer treatment 53. As a regenerative strategy, although the transgene-free approach for chemically generating induced neurons in the injured adult spinal cord still faces many challenges, it holds a promise for clinical applications in the future.

In conclusion, our study gains insights into the molecular basis of astroglial-lineage conversion and provides a chemical approach to reprogram reactive astrocytes into neurons in the injured adult spinal cord.

## Supplementary Material

Supplementary figures and tables.
