# Supplementary material for: NOTCH1 signaling regulates the latent neurogenic program in adult reactive astrocytes after spinal cord injury
Source: Theranostics. 2022 May 27;12(10):4548–63. doi: 10.7150/thno.71378 (PMC9254256; doi:10.7150/thno.71378)
Supplement: Supplementary file 1 — Supplementary figures and tables. [file thnov12p4548s1.pdf]

Supplementary information

NOTCH1 signaling regulates the latent neurogenic program in adult reactive astrocytes after spinal cord injury

Zijian Tan, Shangyao Qin, Yimin Yuan, Xin Hu, Xiao Huang, Hong Liu, Yingyan Pu, Cheng He, Zhida Su

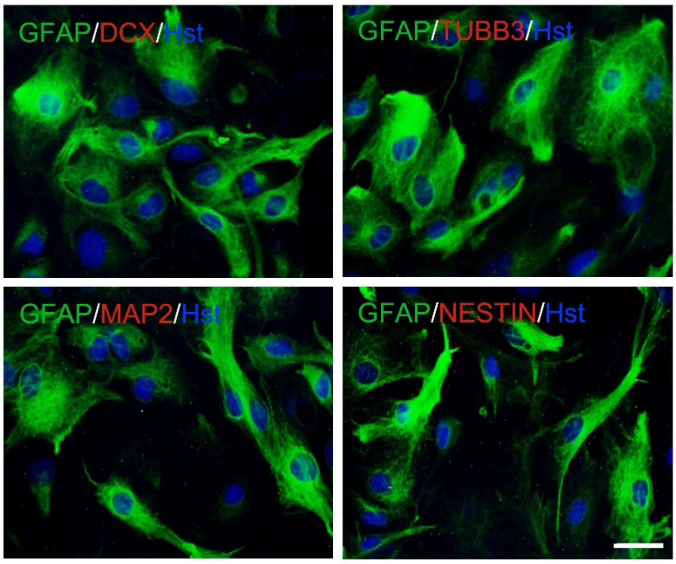

Supplementary Figure S1. Characterization of cultured astrocytes. Cultured astrocytes were immunostained with antibodies against GFAP, DCX, TUBB3, MAP2 and NESTIN. The scale bar represents 25 μm.

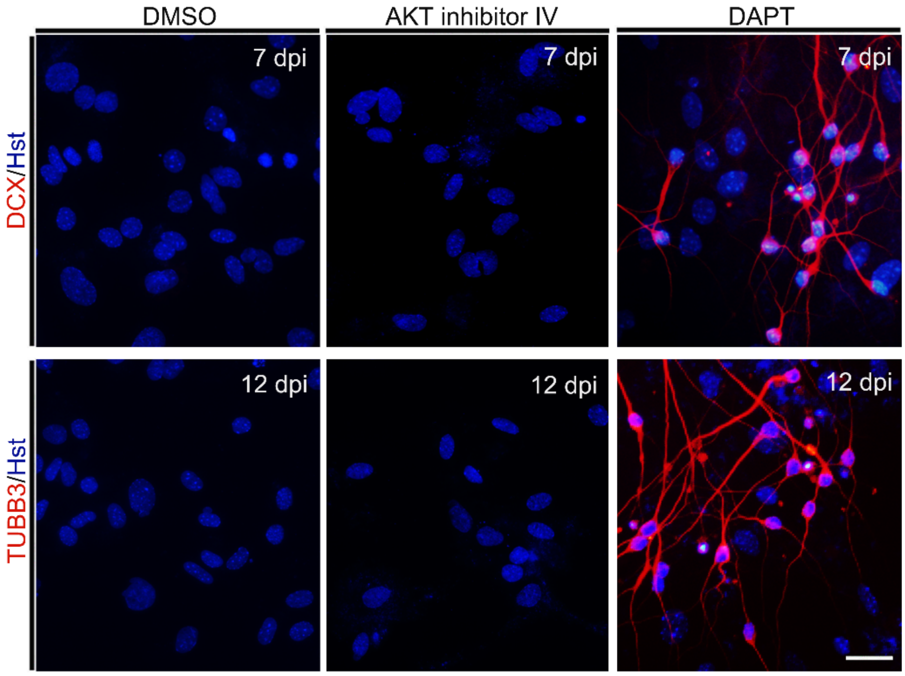

Supplementary Figure S2. Inhibition of AKT signaling fails to reprogram astrocytes into neurons *in vitro*. DCX and TUBB3 immunostaining showed that inhibition of NOTCH1 signaling with DAPT successfully induced astrocyte-to-neuron conversion, whereas inhibition of AKT signaling with AKT inhibitor IV failed to convert cultured astrocytes into neurons. The scale bar represents 25 µm.

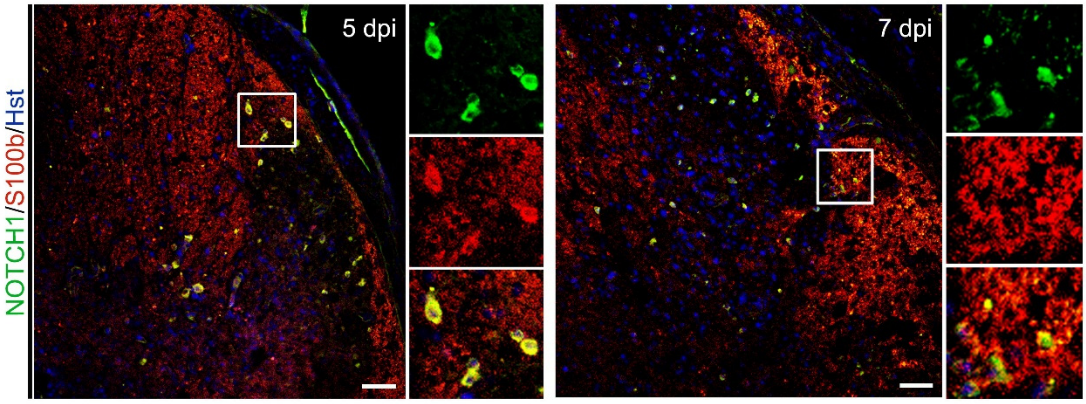

Supplementary Figure S3. Expression of Notch1 in spinal astrocytes after SCI. Immunohistochemical analysis was performed by staining with antibodies against NOTCH1 and S100. The scale bars represent 50 μm

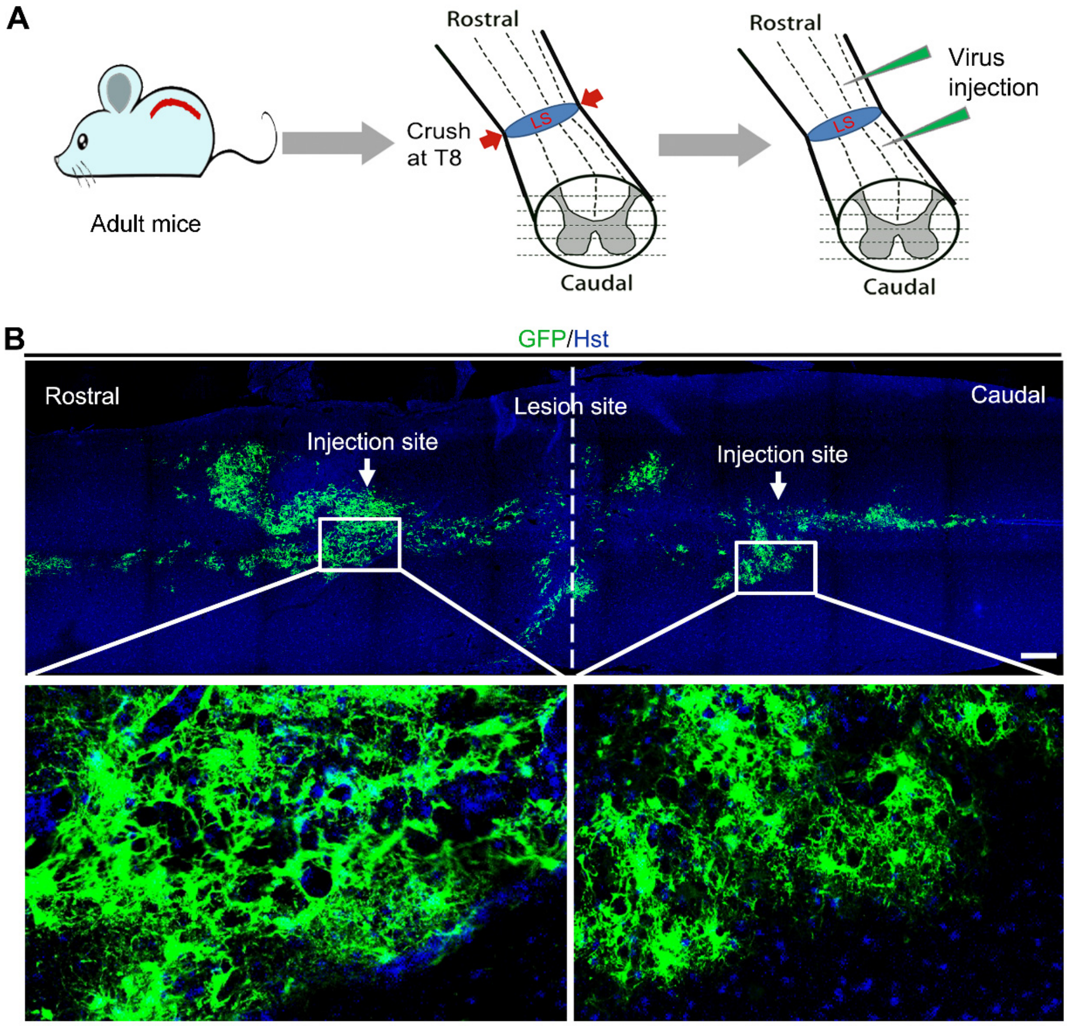

Supplementary Figure S4. SCI model and virus injection. (A) The spinal cord of adult mouse was crushed at T8 level. Lentivirus was manually injected into the spinal cord parenchyma at each of the two locations 1.5 mm apart the lesion site (LS). **(B)** A lower magnification view of a longitudinal section from the adult spinal cord injected with letivirus at 1 wpi. Arrows show the injection sites. The scale bar represents 100 μm.

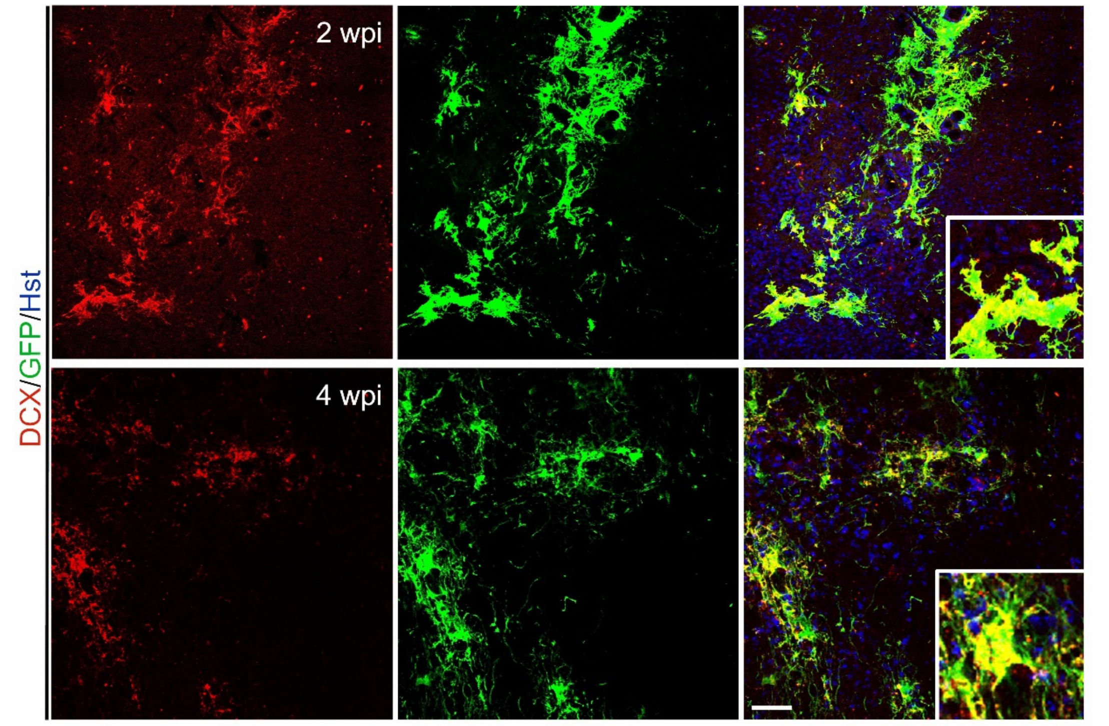

Supplementary Figure S5. Notch1 knockdown in spinal astrocytes triggers neuroblast production in the injured spinal cord. Notch1 in astrocytes was knocked down by hGFAP promoter-driven shRNA. Immunohistochemical analysis was performed by immunostaining with DCX and GFP at 2 and 4 wpi. The scale bar represents 50 μm.

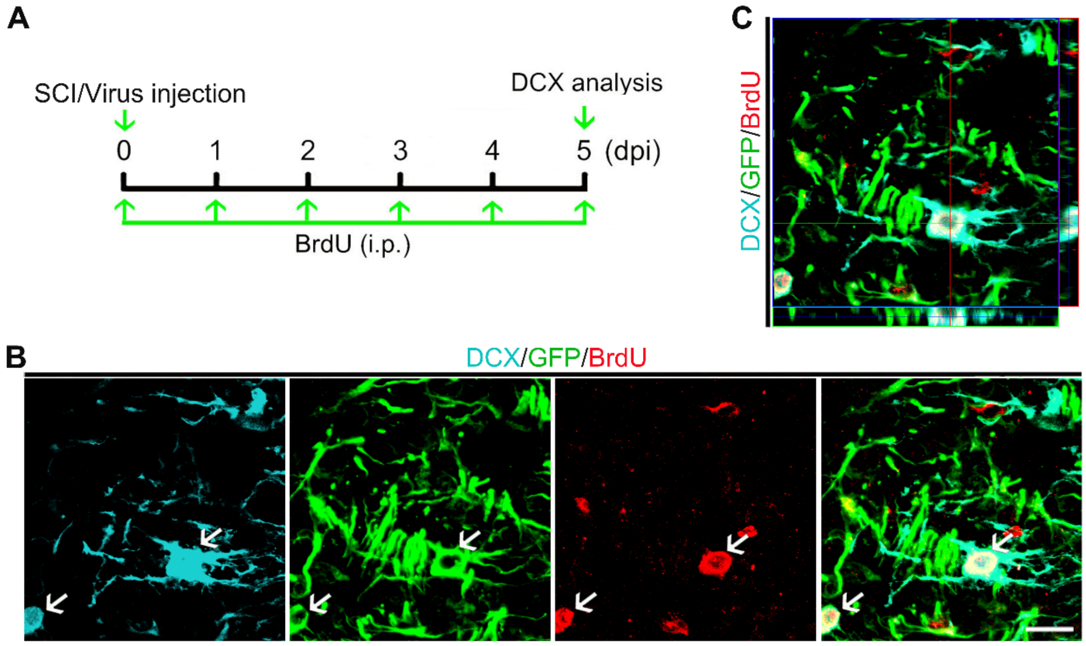

Supplementary Figure S6. BrdU tracing of induced DCX-positive cells. (A) Schematic diagrams of study design for (B) and (C). Lentivirus expressing hGFAP-Notch1 shRNA was injected into crush-injured spinal cord. **(B and C)** Incorporation of BrdU in induced DCX-positive cells. Arrows indicate the $BrdU^{+}/DCX^{+}/GFP^{+}$ cells. An orthogonal view of $BrdU^{+}/DCX^{+}/GFP^{+}$ cells is shown in (C). The scale bar represents 25 μm.

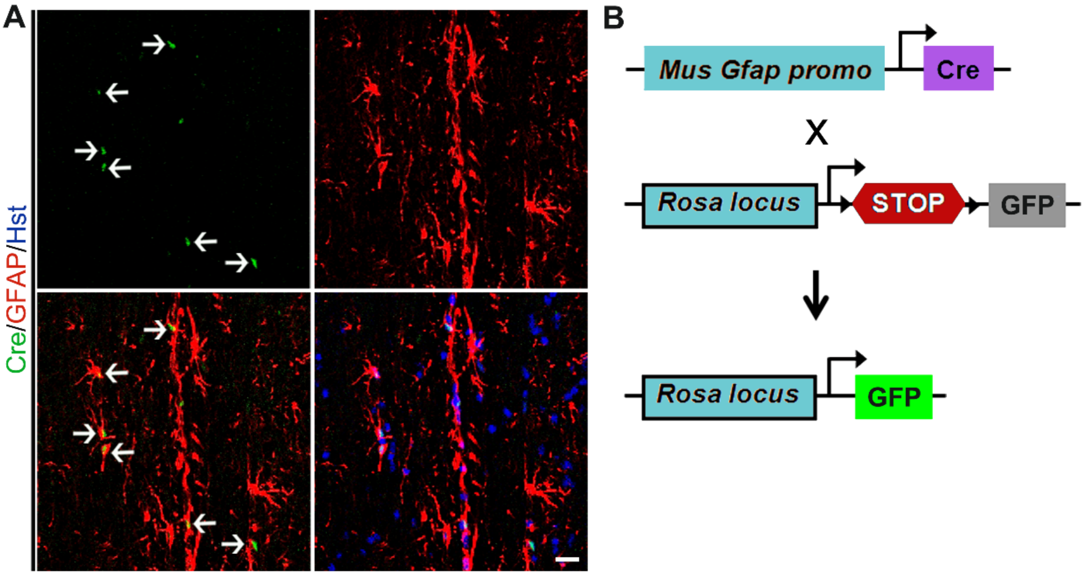

Supplementary Figure S7. Generation of mGfap::Cre; Rosa::GFP transgenic mice. (A) Immunostaining with Cre and GFAP showed that astrocytes were specifically targeted in mGfap::Cre line mice. (B) Schematic diagram of experimental procedures for the generation of mGfap::Cre; Rosa::GFP transgenic mice. The scale bar represents 25 μm.

Supplementary Table S1. Primary antibodies used for western blot

Antibody	Host	Dilution	Source	Catalog number
NOTCH1	Rabbit	1:1000	Cell Signaling Technology	#4147
p-mTOR (Phospho-mTOR)	Rabbit	1:1000	Cell Signaling Technology	#2971
p-STAT3 (Phospho-STAT3)	Rabbit	1:1000	Cell Signaling Technology	#9145
p-AKT (Phospho-AKT)	Rabbit	1:1000	Cell Signaling Technology	#9271
AKT	Rabbit	1:1000	Cell Signaling Technology	#9272
p-P38 (Phospho-p38 MAPK)	Rabbit	1:1000	Cell Signaling Technology	#9211
P38 (p38 MAPK)	Rabbit	1:1000	Cell Signaling Technology	#9212
GAPDH (Glyceraldehyde-3-phosphate dehydrogenase)	Rabbit	1:5000	Proteintech	10494-1-AP
β-ACTIN	Mouse	1:5000	Proteintech	66009-1-Ig

Supplementary Table S2: Primers used for qRT-PCR

Gene	Primer sequence (5'-3')
Notch1	sense: GATGGCCTCAATGGGTACAAG<br>antisense: TCGTTGTTGTTGATGTCACAGT
Hes1	sense: TCAACACGACACCGGACAAAC<br>antisense: ATGCCGGGAGCTATCTTTCTT
Hey1	sense: GCGCGGACGAGAATGGAAA<br>antisense: TCAGGTGATCCACAGTCATCTG
NeuroD1	sense: ACGCAGAAGGCAAGGTGTCC<br>antisense: GTTCCTCGTCCTGAGAACTG
NeruoD2	sense: AAGCCAGTGTCTCTTCGTGG<br>antisense: GCCTTGGTCATCTTGCGTTT
Pax6	sense: TAGCCCAGTATAAACGGGAGTG<br>antisense: CCAGGTTGCGAAGAACTCTG
Lmx1a	sense: ACGGCCTGAAGATGGAGGA<br>antisense: CAGAAACCTGTCCGAGATGAC
Lhx6	sense: CGGCAAGAATATCTGCTCCAG<br>antisense: CAAATCGGCTGAAGTAGTCCAT

Supplementary Table S3. Primary antibodies used for immunostaining

Antibody	Host	Dilution	Source	Catalog number
ALDOC (Aldolase C)	Goat	1:100	Santa Cruz	sc-12065
BrdU (5-bromodeoxyuridine)	Rat	1:100	Abcam	Ab6326
ChAT (Choline acetyltransferase)	Goat	1:100	Millipore	#AB144P
DCX (Doublecortin)	Rabbit	1:400	Cell Signaling Technology	#4604
DCX (Doublecortin)	Mouse	1:100	Thermo	MA5-17066
GABA (Gamma aminobutyric acid)	Rabbit	1:200	Sigma	A2052
GAD6 (Glutamic acid decarboxylase 65/67)	Rabbit	1:200	Abcam	Ab183999
GFAP (Glial fibrillary acidic protein)	Mouse	1:200	Sigma	G3893
GFAP (Glial fibrillary acidic protein)	Goat	1:100	Abcam	Ab53554
GFP (Green fluorescent protein)	Chicken	1:600	Aves Labs	1020
MAP2 (Microtubule-associated protein-2)	Rabbit	1:200	Abcam	Ab32454
NESTIN	Rat	1:100	Santa Cruz	sc-33677
NeuN (Neuron-specific nuclear protein)	Mouse	1:100	Millipore	MAB377
NOTCH1	Rabbit	1:100	Abcam	Ab65297
S100b (S100 calcium binding protein B)	Mouse	1:100	Boster	BM0120
SYN-1 (Synapsin-1)	Rabbit	1:200	Cell Signaling Technology	#5297
TUBB3 (Tubulin beta-3)	Rabbit	1:200	Abcam	Ab18207
TUBB3 (Tubulin beta-3)	Mouse	1:200	BioLegend	801202
VGLUT-1 (Vesicular glutamate transporter 1)	Rabbit	1:200	Synaptic Systems	135302
